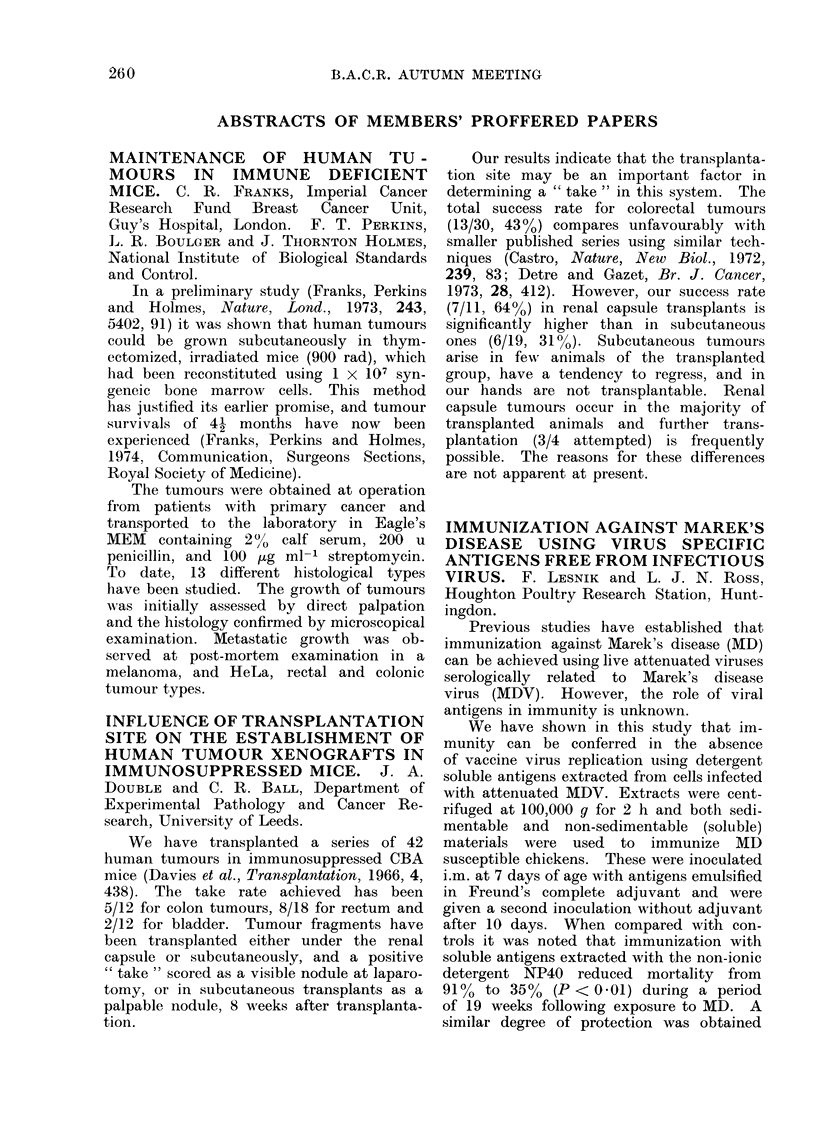# Proceedings: Maintenance of human tumours in immune deficient mice.

**DOI:** 10.1038/bjc.1975.36

**Published:** 1975-02

**Authors:** C. R. Franks, F. T. Perkins, L. R. Boulger, J. T. Holmes


### ABSTRACTS OF MEMBERS' PROFFERED PAPERS

**MAINTENANCE OF HUMAN TUMOURS IN IMMUNE DEFICIENT MICE.** C. R. FRANKS, Imperial Cancer Research Fund Breast Cancer Unit, Guy's Hospital, London. F. T. PERKINS, L. R. BOULGER and J. THORNTON HOLMES, National Institute of Biological Standards and Control.

In a preliminary study (Franks, Perkins and Holmes, *Nature, Lond.*, 1973, **243**, 5402, 91) it was shown that human tumours could be grown subcutaneously in thymectomized, irradiated mice (900 rad), which had been reconstituted using $1 \times 10^7$ syngeneic bone marrow cells. This method has justified its earlier promise, and tumour survivals of $4\frac{1}{2}$ months have now been experienced (Franks, Perkins and Holmes, 1974, Communication, Surgeons Sections, Royal Society of Medicine).

The tumours were obtained at operation from patients with primary cancer and transported to the laboratory in Eagle's MEM containing 2% calf serum, 200 u penicillin, and 100 $\mu$g ml$^{-1}$ streptomycin. To date, 13 different histological types have been studied. The growth of tumours was initially assessed by direct palpation and the histology confirmed by microscopical examination. Metastatic growth was observed at post-mortem examination in a melanoma, and HeLa, rectal and colonic tumour types.